# Parents in the Driver’s Seat—Experiences of Parent-Delivered Baby-mCIMT Coached Remotely

**DOI:** 10.3390/jcm13164864

**Published:** 2024-08-18

**Authors:** Katarina Svensson, Ann-Christin Eliasson, Heléne Sundelin, Kajsa Lidström Holmqvist

**Affiliations:** 1 Division of Children’s and Women’s Health, Department of Biomedical and Clinical Sciences, Linkoping University, 58183 Linkoping, Sweden; helene.sundelin@ki.se; 2 Crown Princess Victoria’s Children Hospital, 58185 Linkoping, Sweden; 3 Neuropediatric Unit, Department of Women’s and Children’s Health, Karolinska Institute, 17177 Stockholm, Sweden; ann-christin.eliasson@ki.se; 4 Neuropaediatric Research Unit, Astrid Lindgren Children’s Hospital, 17176 Stockholm, Sweden; 5 University Health Care Research Center, Department of Neurology and Rehabilitation Medicine, Faculty of Medicine and Health, Örebro University, 70182 Örebro, Sweden; kajsa.lidstrom-holmqvist@regionorebrolan.se

**Keywords:** early intervention, remote Baby-mCIMT, telerehabilitation, unilateral cerebral palsy, home-training, parent experience

## Abstract

**Background/Objectives**: Recent guidelines on early intervention in children at high risk of cerebral palsy (CP) recommend parental involvement and family-centered home-based interventions with parents as primary trainers. Therapist coaching by home visitation is resource demanding, and telerehabilitation is a viable option for remote intervention and coaching. This study aims to describe parents’ experiences of engaging in Baby-mCIMT coached remotely. Their infants are at high risk of unilateral cerebral palsy and the parents have been the primary trainers in regard to home-based intervention, optimizing the use of the affected hand. **Methods**: A qualitative approach involving semi-structured interviews with eight parents was employed. Data were analyzed using qualitative content analysis. **Results**: The overarching theme “Parents in the driver’s seat—learning through remote coaching to create conditions to enhance the child’s motor skills” describes parents’ experiences as primary training providers. The following three underlying categories with subcategories were identified: (1) Baby-mCIMT coached remotely in an everyday context—practical and technical prerequisites; (2) the child’s response and the therapists’ coaching supports active parental learning; (3) capability and sense of control—strengthening and demanding aspects. **Conclusions**: Our findings revealed that Baby-mCIMT coached remotely empowered the parents as primary trainers, which provided them with opportunities for understanding and learning about their child and their development. The findings underscore the importance of responsive professional guidance and a strong therapist–parent relationship to succeed with the Baby-mCIMT program coached remotely and to manage the digital coaching format.

## 1. Introduction

Recent guidelines for early intervention in children at high risk of cerebral palsy (CP) recommend parental involvement and family-centered approaches within enriched environments [1]. Home-based interventions with parents as primary trainers are advocated [2]. Telerehabilitation is promoted as a family-centered approach [3], with previous studies indicating positive perceptions of families engaged in telerehabilitation [4,5]. Constraint Induced Movement Therapy (CIMT) has shown effectiveness in improving motor skills in the affected hands of children with unilateral CP by restraining the non-affected hand [6]. CIMT has been adapted for infants (Baby-mCIMT), starting at approximately four to five months of age, and has shown efficacy in improving motor skills in the affected hand [1,7,8]. This intervention promotes self-initiated hand movements through tailored repetitive activities that involve the restraint of the non-affected hand and high-intensity training over a limited period. In Baby-mCIMT, parents conduct home-based training with coaching and supervision from therapists once a week [9]. Remote Baby-CIMT, the digital version of Baby-mCIMT, was introduced at a habilitation center in Stockholm in 2016. In this approach, therapists’ home visits are replaced with web camera meetings. Our recent study showed comparable effects on hand function when using remote coaching versus in-person coaching with Baby-mCIMT [10]. Overall, research on parents’ experiences as primary training providers in a digital context is limited. However, in a study by Verhaegh et al. [11], parental experiences with remote coaching indicated that successful implementation requires support for parents due to several challenges and the high parental load that needs to be considered.

Successful coaching of parents relies on establishing a trusting relationship among the therapist, child and parents to enable the parents to gain comprehensive knowledge about their child and actions needed to facilitate its development [12,13,14,15]. However, parents’ ability to engage in early interventions may be affected by the new, complex and often stressful challenges of having a child affected by a brain insult [16]. Therefore, it is crucial to consider parental stress and its impact on well-being along with parents’ abilities and experiences in daily training [17,18,19]. Research on parental perspectives is limited, and deepening the comprehension of parental perspectives within a digital context is vital for successful interventions, as well as for further development of such interventions. Therefore, this study aims to describe parents’ experiences in engaging with Baby-mCIMT coached remotely for infants at high risk of unilateral CP.

## 2. Materials and Methods

### 2.1. Design

This study had a qualitative design where data were collected through semi-structured interviews with parents who had participated in Baby-mCIMT coached remotely. Qualitative content analysis was used for data analysis [20]. The COREQ checklist [21] was utilized to ensure transparent and comprehensive reporting. The remote Baby-CIMT studies were approved by the Stockholm Regional Ethical Review Board, Sweden (dnr 2015/1571-31/4), with an amendment for parental interviews approved by the Swedish Ethical Review Authority (dnr 2021-06774-0).

### 2.2. Baby-CIMT in a Remote Digital Coaching Context

The principles of Baby-mCIMT with in-person coaching have been described previously [9]. Remote-Baby-mCIMT follows the same principle but with parents being coached in a remote digital context. It is an intervention designed to promote development in the affected hand for children at risk of unilateral CP [9]. The infants offered the intervention are selected based on neurological signs and asymmetric hand functions measured by Hand Assessment for Infants, although a definitive diagnosis may not have been confirmed. The intervention involves intensive activity-based training by restraining the non-affected hand with a mitten, clip or similar device accepted by the child. The regime consists of 36 h in total, which is divided into 30 min parent-led training sessions held 6 days a week for two 6-week periods, separated by a 6 week break. As a start-up for the intervention, one home visit is included in the intervention to strengthen the relationship between the therapist and the family. Thereafter, the parents receive supervision and coaching from the therapist through digital meetings at a training session once a week. The therapists in the study worked at a special unit for children with special needs. Baby-mCIMT coached remotely also consists of web-based materials that are accessible through a web platform, including instructions, information and inspirational content [10]. The parents play a central role in this intervention, as they serve as the primary trainers, and their active engagement in providing the daily training is essential for its effectiveness. However, arranging and organizing playful training, as well as education, supervision and coaching of parents by an experienced therapist, is crucial for success. The coaching approach focuses on the parents’ self-efficacy and empowerment to implement the intervention through playful activities tailored to the child’s functional level. It includes supporting and guiding parents as problem solvers to find various solutions for different kinds of playful practices. It is important to strengthen parents in their roles as experts on their child. Goals are collaboratively set and recorded in a diary, serving as a focus area for the upcoming week.

### 2.3. Participants

All parent couples (*n* = 10) with infants aged 4–10 months (corrected age) at high risk of unilateral CP who participated in the Baby-mCIMT coached remotely between 2020 and 2022 were invited to participate. These parent couples received an information letter with a consent form via mail followed by a phone call to address any questions. Eight parents from seven families consented to participate. One parent couple was interviewed together, one parent couple declined participation for family reasons, and two parent couples did not respond to the invitation letter. Participant characteristics are presented in Table 1. This study was conducted in Sweden, where all parents are entitled to paid parental leave during the child’s first year, ensuring that one parent from each couple was on parental leave during the intervention.

### 2.4. Interview Guide

A study specific semi-structured interview guide was developed by Author 1 and 2. Author 1 is a female PhD student and physiotherapist with previous experience of semi-structured interviewing and of providing the intervention. Author 2 is a female professor and occupational therapist with extensive experience in conducting Baby-mCIMT who also contributed to the guide. The interview guide covered broad areas related to the study’s objectives, using probing questions to delve deeper into each area. The areas explored participants’ experiences regarding the intervention itself, its potential impact, challenges or opportunities that were encountered, and the participants’ roles as training providers. Although no pilot interviews were conducted, after the first interview Authors 1 and 2 discussed the interview guide and determined that no refinements were necessary. Consequently, data collection commenced with the first interview.

### 2.5. Data Collection

The interviews were conducted by the first author between March and June 2022. She had no prior relationship with the participants. The interviews were performed within a time frame ranging from 2 to 22 months (Md = 4) after finishing the intervention. The parents had the option to be interviewed either in-person or via video calls and all opted for the latter. In two interviews, the child was present in the background; apart from that, only the parent and the interviewer were present. Before the start of the interview, the interviewer repeated the reasons for conducting this research study and eventual questions were answered. The interviews lasted between 35 and 48 min (Md = 38). No interviews were repeated or supplemented. Each interview was audio-recorded and transcribed verbatim by the first author. To ensure anonymity, all names were removed from the transcripts. Transcripts were not returned to the parents for comments.

### 2.6. Data Analysis

Inductive qualitative content analysis in accordance with Graneheim and Lundman [20] was used for analysis. The Qualitative Data Analysis Software NVivo (version 1.7.1) was used for organizing the data, coding and data visualization.

Initially, all transcripts were extensively reviewed by the first and last authors to familiarize themselves with the data and gain an overall understanding of the content. The last author, a female associate professor, occupational therapist and experienced qualitative researcher, possessed expertise in content analysis but had not been previously involved in the intervention or had any prior relationship with the participants.

Subsequently, the first author divided the texts into meaning units, representing portions of text describing similar content. These units were further condensed while preserving the essential content. Each condensed unit was then assigned a code reflecting its content at a manifest level, characterized at a low level of abstraction. The condensed meaning units and codes were then discussed until consensus between the first and last author was achieved. The analysis continued with a categorization process wherein codes were compared for similarities and differences iteratively, leading to a preliminary categorization of subcategories. This tentative categorization was scrutinized and discussed several times between the first and last author, resulting in further categorization at a higher level of abstraction wherein subcategories were grouped into categories based on their content. An overarching theme was formulated connecting all categories at an even higher level of abstraction. The preliminary findings were discussed by all authors. Author 3, a female associate professor and pediatric neurologist with expertise in interacting with children and parents eligible for Baby-mCIMT, provided additional insights during joint discussions. During the joint discussions, some adjustments to the categorization were made and the findings were agreed upon, with the descriptive overarching theme being “Parents in the driver’s seat—learning through remote coaching to create conditions to enhance the child’s motor skills” along with three underlying categories and subcategories. An example of the analysis process is found in Table 2.

## 3. Results

**Figure 1:**
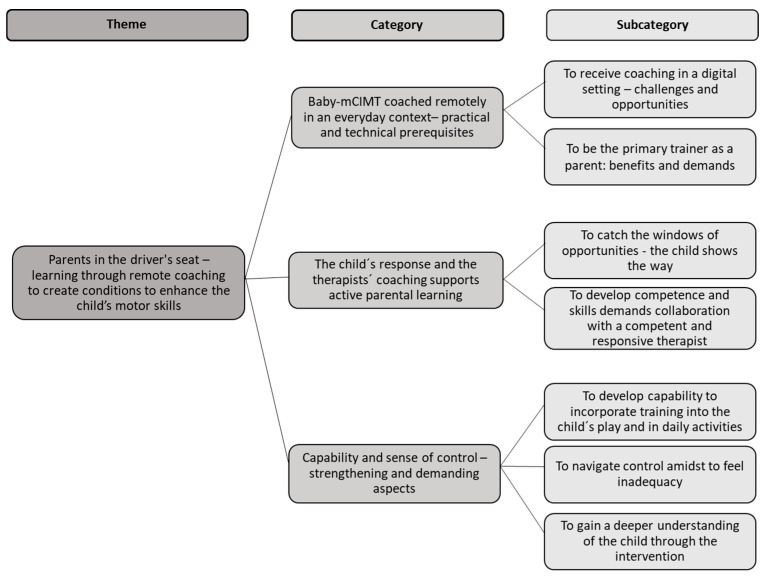
Theme, categories and subcategories.

The overall theme is “Parents in the driver’s seat—learning through remote coaching to create conditions to enhance the child’s motor skills”. The theme illustrates the parents’ learning journey, wherein they, supported by digital coaching, acquired knowledge, skills and competence to train their child’s hand function by participating in Baby-mCIMT coached remotely. While assuming the pivotal role of training providers, the parents found the role both natural and demanding at the same time, as they had to navigate various challenges. Further, they described how their own development of capabilities instilled a sense of control in their otherwise uncertain journey. Parents’ experiences with remote Baby-CIMT are described in the following three main categories: “Baby-mCIMT coached remotely in an everyday context—practical and technical prerequisites”, “The child’s response and the therapists’ coaching supports active parental learning” and “Capability and sense of control—strengthening and demanding aspects”. The theme with underlying categories and subcategories is displayed in Figure 1.

### 3.1. Baby-mCIMTcoached Remotely in an Everyday Context—Practical and Technical Prerequisites

In this category, the parents shared their experiences of Baby-mCIMT coached remotely along with important technical and practical aspects for its implementation. The parents underscored the benefits of integrating the intervention into everyday life but also noting its time-consuming and demanding nature. Despite challenges, the parents ultimately found the intervention worthwhile. This is further elaborated in the subcategories “To receive coaching in a digital setting—challenges and opportunities” and “To be the primary trainer as a parent: benefits and demands”.

#### 3.1.1. To Receive Coaching in a Digital Setting—Challenges and Opportunities

In this subcategory, the parents described their perceptions of the video meetings, influenced by their child’s motivation, their own attitude, the technical quality and the therapist’s approach and communication skills. Some parents described the digital coaching sessions as being easy to implement, while others found them more challenging. As one parent said:

“You just put the tablet on the table, and then you work as usual. So, it wasn’t something I thought would be limiting; on the contrary, it became more convenient for us parents. We don’t have to go somewhere, which takes time even if we live nearby.” (1)

However, the parents described variations in their child’s participation. Some parents found the sessions successful, while others were presented with challenges in actively engaging the child in training activities in front of the screen.

The parents utilized the video meetings in various ways. Some described them as being beneficial for displaying progress and receiving further guidance and feedback on how to proceed. Others expressed that they faced challenges in taking full advantage of the therapist’s expertise in the digital setting, especially when their child’s participation and motivation were low. As one parent put it:

“You could still kind of sense that he knew he had to display what he can do in some way, so he wasn’t always super enthusiastic about doing it right then.” (5)

The parents emphasized that the effectiveness of the digital meetings depended on the therapist’s ability to observe the child’s responses and be sensitive to the parents’ situation and communication skills. It was expressed that the video meetings aroused intense emotions due to the pressure to perform well and demonstrate progress to the therapist.

Technical issues were generally not perceived as a significant drawback, and they were mostly experienced as manageable. The parents found alternative solutions or had supplementing digital meetings with phone calls if needed.

#### 3.1.2. To Be the Primary Trainer as a Parent: Benefits and Demands

In this subcategory, the parents shared their general experiences of being the primary trainer. Many of the parents found the concept of Baby-mCIMT strenuous and time-consuming. Home-based training was described as natural and adaptable to the child’s needs and could be seamlessly integrated into daily life. Some parents described the training as straightforward, while others encountered challenges, particularly when the activities were perceived as being too difficult for their child. It also allowed for shared responsibility between the parents and sometimes both parents participated in digital meetings, although typically the parent on parental leave was described as having the primary role. The parents expressed appreciation for having the intervention during their child’s first year despite it being a busy and challenging period due to medical complications and frequent healthcare visits. As one parent remarked:

“I think it is good that we did it … not waited until he was a bit older, it was a bit calmer to even do it in that business… the first bit was tough cause it was like just a tough time in general.” (7)

In retrospect, the parents acknowledged that the Baby-mCIMT coached remotely was demanding. However, upon reflection, they recognized its effectiveness. Despite potential challenges, the parents expressed positive experiences with the intervention and noted positive training results for their child, making them believe it to be worthwhile. They appreciated participation in a proven method that provided structured and well-adapted regular support.

### 3.2. The Child’s Response and the Therapists’ Coaching Supports Active Parental Learning

In this category, the parents described how they have learned to identify and adapt to the child’s “windows of opportunities”. They learned to identify the opportune moment when their child was most likely to succeed and capitalized on it to create ideal conditions for engaging in playful training. In addition, they noted that therapists, with their expertise and responsiveness, had facilitated their engagement in the intervention, which enhanced their awareness and understanding of their child’s progress. All aspects together deepened their competence and skills. This is described in more detail in the subcategories “To catch the windows of opportunities—the child shows the way” and “To develop competence and skills demands collaboration with a competent and responsive therapist”.

#### 3.2.1. To Catch the Windows of Opportunities—The Child Shows the Way

In this subcategory, the parents delineated key factors for successful training. They highlighted the importance of both routines and flexibility. They described how they utilized the child’s “windows of opportunities”, meaning to choose playful and creative training aligning with the child’s interests and abilities at a time when the child was most receptive. Favorable practice times were typically in the morning when the child was well rested and fed. Training durations varied, ranging from the expected 30 min to as short as 5 min depending on the child’s endurance. Challenges arose when the child resisted wearing the mitten or clips on the unaffected hand, sometimes necessitating the parents to remove the restraint to be able to continue the training of the affected hand.

The parents described that, when they found the right conditions or moments for training, they observed the child’s willingness to engage its affected hand during play. As one parent remarked:

“… a little surprised that it went so well and if you make it into play, it works” (3)

They stressed the importance of play and noted that, if play does not work, neither does training. However, the ability to integrate play into training varied among the parents; some found it natural, while others struggled to make it fun and to maintain the child’s interest and focus. The parents also described the importance of adjusting the play to the child’s level and to ensure a variety of playful activities to maintain the engagement of both the child and themselves. They emphasized the significance of customized and varied toys, focusing on enjoyment rather than achievement.

#### 3.2.2. To Develop Competence and Skills Demands Collaboration with a Competent and Responsive Therapist

In this subcategory, the parents expressed the importance of receiving professional guidance from a competent and responsive therapist, which they deemed crucial for developing their skills and competence in delivering effective training. They appreciated the therapist’s expertise in child development, an area where they, as parents, often felt a lack of knowledge. The therapists’ expertise provided them with insights into expected progress and offered advice on training direction and implementation. The parents also emphasized the importance of receiving guidance in problem-solving, skill development, and monitoring their child’s progress. They appreciated the therapist’s provision of ideas, inspiration, and guidance for the training. Several parents acknowledged that the therapists have skills and techniques with children that they themselves lack. As one parent remarked:

“I guess what I’m trying to say is that it takes some skill from the parents to be able to do this in a good way and I realized that I didn’t have that skill so I had to try to develop it…” (4)

To manage their role as primary trainers, the parents highlighted the importance of a strong relationship with the therapist. They emphasized the therapist’s sensitivity to their circumstances and their need for reassurance and encouragement, particularly in alleviating feelings of not doing enough. The parents emphasized the therapist’s responsiveness to the child’s daily condition during digital meetings. They also highlighted the therapist’s support in acknowledging small steps of progress and alleviating feelings of failure. Additionally, the parents described the importance of therapists being aware of the parents’ life situation, their anxiety regarding the child’s development, as well as their individual preferences in terms of receiving information along with their understanding of their role as primary trainers. The parents expressed gratitude for the opportunity to participate and appreciated the support provided by professionals. As one parent expressed:

“Just being in the driver’s seat, and getting this coaching and support, you don’t feel so alone… that’s what I learned… that there is still… people who are there for you… and it felt really good, I think. You also learn a lot about yourself, so you can get through it if you help each other out.” (6)

### 3.3. Capability and Sense of Control—Strengthening and Demanding Aspects

In this category, the parents described various aspects of gaining a sense of control. The parents highlighted several strengthening aspects following participation in Baby-mCIMT coached remotely. These aspects included gaining the capability to apply training principles in play and to integrate training into daily activities along with a deeper understanding of the child. On the other hand, they also described demanding aspects tied to their capabilities and sense of control, such as the stress of being solely responsible for the training, which caused feelings of performance pressure. This is further elaborated in the subcategories “To develop capability to incorporate training into the child’s play and in daily activities”, “To navigate control amidst feelings of inadequacy” and “To gain a deeper understanding of the child through the intervention”.

#### 3.3.1. To Develop Capability to Incorporate Training into the Child’s Play and in Daily Activities

In this subcategory, the parents recognized the importance of observing how their child learns to use the hand in everyday activities in a natural manner. The parents described how they used the therapist’s advice of using simple toys found at home as training material. Further, they expressed how they had learned to integrate the training principles into daily activities, which they acknowledged as a strength. They utilized their capability to engage the child in training and identify suitable toys for skill development. As one parent said:

“… And maybe it was when we understood, you know, when we got a bit into it and grasped what he needed to practice, that’s when we could try, like, “Okay, let’s try this grip… you can try with this object, well, it’s kind of similar.” Yeah, it requires understanding what he needs to practice and how that grip should be exactly to get it right…” (5)

#### 3.3.2. To Navigate Control Amidst Feelings of Inadequacy

In this subcategory, the parents expressed a sense of control alongside the stress of being responsible for their child’s development, which was found to be demanding and challenging. They strived to do their best for their child, but they also grappled with feelings of performance pressure, as one parent articulated:

“How can we not manage to get 30 min (training)? It’s not okay, that we’re not giving her that”… so that was maybe the downside of being in control, that sometimes it just didn’t work out.” (6)

The parents also highlighted the importance of maintaining balance and not allowing the training to constantly occupy their thoughts. They described sharing their feelings with the therapist as one way to alleviate the pressure of performance. As one parent described:

“That one session when I said to the therapist or just like just trying really hard cause I think they just made loads of making loads of suggestions and I was I kind of feel like I am doing all these things you know that made me feel like I was failing you know.” (7)

Undertaking the roles of both parent and training provider offered an opportunity for understanding, acceptance and empowerment. The parents described various aspects of taking control, including transitioning from denial to acceptance in regard to their child’s limitations and being able to communicate with their support network. There were also descriptions of how the training brought attention to their own emotions and heightened their self-awareness.

#### 3.3.3. To Gain a Deeper Understanding of the Child through the Intervention

In this subcategory, the parents expressed how they gained a deeper understanding of their child’s development and learned to adapt to their child’s desires, needs and functioning, even when others may not notice. As one parent stated:

“Like now, even when you go to the doctors and they’re checking if he’s developing as he should, even if they don’t see certain things, I know and I feel confident in what I observe” (2)

The parents described how they had become more attentive to what to look for, enhancing their ability to interpret their child’s behavior and to tailor the training based on their child’s abilities. They felt challenged in regard to providing training adapted to specific areas of focus, recognizing small improvements, and understanding how tasks could be modified.

Some concerns were expressed at the end of the intervention regarding aspects such as how to proceed, opportunities for additional intensive training, the responsibility of expressing training preferences to healthcare providers and whether the continued training responsibility lies with the family.

## 4. Discussion

The main findings of our study showed that parents undergo a learning process as they develop skills in terms of training provision. Considering the significant impact that parents’ abilities have on their child’s progress, it is essential to prioritize their learning and skill development when designing home training interventions. This finding is in line with previous studies emphasizing the importance of parental learning in interventions for children with disabilities [22,23]. Parents’ learning processes can be understood based on theories regarding adult education [24,25] that state that learning typically occurs through problem-focused experiences. According to these theories, the acquisition of practical knowledge, with opportunities for self-directed learning and internal rewards, enhances self-confidence and motivation [24]. Additionally, McCarthy and Guerin [26] suggest that, in early interventions, parents’ learning is initiated by their ability to absorb the therapist’s skills and active engagement in their child’s development, which is in line with the findings of our study.

The coaching provided by therapists in pediatric rehabilitation has been shown to play a vital role in achieving success, and it has also been emphasized in a framework of solution-focused coaching that involves parents’ reflections to effectively transfer knowledge and capability to their child’s natural environment [27]. Our findings demonstrate that parents’ abilities to implement the therapist’s advice into practice can be supported in a digital context. The parents in our study expressed appreciation for the feedback they received from the therapists, which strengthened their role as the primary trainers of their child. Involvement in Baby-mCIMT coached remotely enhanced the parents’ reflective abilities, which has been described as a crucial aspect of the learning process and competence development in early interventions [28].

In our study, the parents found themselves navigating a new life situation while simultaneously assuming a central role in early intervention, where the digital approach required a significant parental commitment. To motivate and empower themselves as primary trainers, they highlighted the importance of a trusting relationship with the therapist. The parents appreciated the therapists’ competence, professionalism and sensitivity to both the child and the parents’ circumstances. The importance of the therapist–parent relationship is confirmed in previous research [14,26], and a high-quality therapist–parent–child relationship has been described as fundamental for parental engagement and successful implementation of early interventions [13,15]. Further, the involvement of therapists in the parents’ situation has been shown to be of great importance [16]. Our findings are in line with these findings and contribute to the growing body of evidence highlighting the importance of the therapist’s responsiveness to the child’s and parents’ communication, which supports parental self-efficacy, learning and sense of control.

The findings of our study demonstrate how the parents’ experience heightened their sense of control, although they also did occasionally grapple with ambivalence. The parents expressed that implementing training daily places high demands on their flexibility and creativity yet instills confidence in their abilities and their understanding of what is best for their child. However, they also described feelings of stress stemming from concerns about not doing enough or not doing the right things, especially when the child’s development progressed slowly or when the parents perceived minimal development. These experiences are confirmed by findings from other studies focusing on early intervention for children at high risk of cerebral palsy [13,15,16]. The feelings of self-doubt are also consistent with the concept of parental self-efficacy, which refers to confidence in one’s own competence or ability to succeed in the parental role [29]. Studies have shown that parental self-efficacy correlates with their understanding of child development and affects factors such as empowerment, positive parent–child interactions and favorable outcomes for the child’s development and well-being [29,30]. Therefore, it is crucial for therapists to ensure that parents’ confidence in their own abilities is not negatively impacted if the child’s condition does not improve.

### Methodological Considerations

To strengthen the trustworthiness of the study, several important aspects were carefully addressed, including credibility, dependability and transferability [20]. The COREQ checklist [21] provided guidance to ensure transparent and comprehensive reporting.

The interview guide was developed by two researchers experienced in providing Baby-mCIMT coached remotely and who were thus familiar with parents’ roles as training providers. However, no pilot interviews were conducted, which could be considered a limitation, as input from parents themselves may have enhanced the credibility of the interview guide. On the other hand, using a predefined interview guide enhanced the dependability of the interviews, and the interviewer made efforts to deepen the parents’ responses through prompts, thereby contributing to the credibility of the collected data. The timing of the interviews varied, with some parents having recently completed the intervention and for others a longer period had passed. This discrepancy in timing of the interviews could potentially affect the parents’ ability to recall their experiences, thereby impacting the credibility of the findings. On the other hand, the parents who had a longer interval since the intervention were able to reflect on their experience with a different perspective, which might have added valuable insights and thus enhanced credibility.

The sample size of eight parents may be perceived as a limitation. However, upon transcribing the last interview, the data were considered to be rich and diverse in content. The last interviews did not add any significant new information and perspectives, and the research group concluded the data collection process, as further interviews were unlikely to yield additional insights. All parents chose to be interviewed digitally in a video meeting, potentially influencing the quality of the interviews by limiting opportunities to interpret non-verbal cues and build rapport with the parents. Nonetheless, given the participants’ familiarity and comfort with video meetings from the previous intervention, this format was deemed satisfactory. In addition, research on digital interviewing in qualitative studies suggests high participant satisfaction, citing convenience and simplicity [31].

It is important to note that Baby-mCIMT coached remotely was carried out in a Swedish context, where parents have the option of paid parental leave, affording them more time to dedicate to the intervention compared to situations where both parents are working. This, and other contextual factors, should be considered when interpreting the transferability of the findings to countries and settings with a different context. In addition, it is important to take the remote context into account when judging transferability, as parents’ experiences of Baby-mCIMT delivered in-person may differ.

Challenges and feelings of stress related to being both the parents and trainers of their children were expressed by some parents. Factors concerning the parents’ mental health state likely influence their ability to cope with their situation and ability to be a training provider. The importance of taking parental stress into consideration has been highlighted in previous studies [18,19], and, even though it was not the primarily focus in this study, the results showed that the parents were overall satisfied with the support provided by their therapist. However, this is an important area for further research.

## 5. Conclusions

The findings on parental experiences with the Baby-mCIMT early intervention program, which was remotely coached, reveal both opportunities for skill development and avenues for parental empowerment. However, parents also expressed feelings of stress and a need for greater flexibility, highlighting the complex dual role they play as both parent and primary trainers. These insights underscore the importance of responsive professional guidance and active engagement, as well as the critical role of a strong therapist–parent relationship in shaping parents’ perceptions of success.

## Figures and Tables

**Table 1:** Participant characteristics.

Parents’ Sex	Age at Start of Intervention (Months)	Time between Intervention and Interview (Months)	Language Spoken During the Interview
Female	6	22	Swedish
Female	4	10	Swedish
Male	7	2	English
Female	7	10	Swedish
Female/Male (couple)	10	4	Swedish
Female	8	3	English
Male	6	4	Swedish

**Table 2:** Example of the analysis process.

Meaning Unit	Code	Sub Category	Category	Theme
And you think that maybe it does not affect that much when it’s on a screen, but it does, voices and like yes how to show movements and so on	The therapist’s responsiveness for reactions and participation	To receive coaching in a digital setting—challenges and opportunities	Baby-mCIMT coached remotely in an everyday context—practical and technical prerequisites	Parents in the driver’s seat—learning through remote coaching to create conditions to enhance the child’s motor skills
But sure that she was under this umbrella feels better for us as parents to feel that we are in the care carousel and that it is supervised through a proven method, yeah that felt much better	Organized method provides structure and well-adapted and regular supervision	To be the primary trainer as a parent: benefits and demands		
And some days are harder than others, and you can’t explain why	The child’s well-being and daily condition affect the training	To catch the windows of opportunities—the child shows the way	The child’s response and the therapists’ coaching supports active parental learning	
Like help being guided by other people, so different ways of learning the skills necessary to train your child	Parents lack the therapist’s expertise—need coaching	To develop competence and skills demands collaboration with a competent and responsive therapist		
They also like objects so everything that we have in the kitchen drawers like different types of cutlery or spoons that you use for cooking	Stimulating and developing training toys	To develop capability to incorporate training into the child’s play and in daily activities	Capability and sense of control—strengthening and demanding aspects	
And I think it felt very good and you learned much about yourself too	Observe feelings and achieve increased self-awareness	To navigate control amidst to feel inadequacy		
And then I thought that you just learned in general about her condition and her limitations	Learned to know the child, understand more of the child’s development, what the child likes and how it functions	To gain a deeper understanding of the child through the intervention		

## Data Availability

Data are unavailable due to ethical restrictions.
